# Programmable RNA targeting with CRISPR-Cas13

**DOI:** 10.1080/15476286.2024.2351657

**Published:** 2024-05-19

**Authors:** Peiguo Shi, Xuebing Wu

**Affiliations:** Department of Medicine and Department of Systems Biology, Columbia University Irving Medical Center, New York, NY, USA

**Keywords:** CRISPR-Cas13, RNA, collateral activity

## Abstract

The RNA-targeting CRISPR-Cas13 system has enabled precise engineering of endogenous RNAs, significantly advancing our understanding of RNA regulation and the development of RNA-based diagnostic and therapeutic applications. This review aims to provide a summary of Cas13-based RNA targeting tools and applications, discuss limitations and challenges of existing tools and suggest potential directions for further development of the RNA targeting system.

## CRISPR-Cas13 as a programmable RNase

The clustered regularly interspaced short palindromic repeats (CRISPRs) and CRISPR-associated (Cas) proteins, are bacterial adaptive immune systems, most of which are potent DNA/RNA endonucleases. In class 2 type VI CRISPR/Cas systems, single-subunit Cas13 system as an RNA-guided RNA endonuclease (RNase) has been reported since 2016. Cas13a (also known as C2c2) is the first subtype of Cas13 family [1–4]. The Cas13 family now comprises 11 subtypes, including Cas13a [1,2], Cas13b [3], Cas13c [4], Cas13d [4], Cas13e [5], Cas13f [5], Cas13g [5], Cas13h [5], Cas13i [5], Cas13x [6] and Cas13y [6], with potentially more to be identified. While these Cas13 systems are all RNA-guided RNases, they differ in size, protein sequence and in their efficiency in eukaryotic cells [5,6]. Among them, Cas13a, Cas13b and Cas13d are the most widely used in mammalian cells.

**Figure 1.**
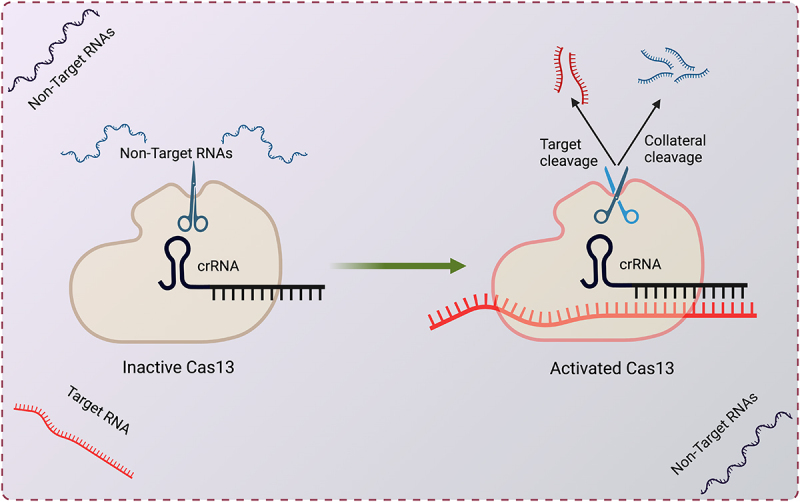
CRISPR-Cas13 and its collateral activity.

Cas13 is guided by a CRISPR RNA (crRNA) and is programmed to cleave RNA targets carrying complementary protospacers [1] (Figure 1). Unlike most DNA-targeting CRISPR/Cas systems that require a protospacer adjacent motif (PAM), Cas13 shows no strong bias on protospacer flanking sequences (PFS) in eukaryotic cells, allowing it to target essentially any sequences. Cas13 proteins typically contain two higher eukaryote and prokaryote nucleotide-binding (HEPN) RNase domains, which form a single catalytic site that cleaves target RNAs [7–10]. Cas13 also possesses another RNase activity that can process its own crRNAs from a pre-crRNA array that consists of multiple repeats of spacers and direct repeats (DRs) [2]. The pre-crRNA processing activity facilitates multiplexed targeting by expressing multiple crRNAs (also called guide RNAs or gRNAs) from a single transcript [11].

Upon recognition of the target RNA by the crRNA (black), the activated Cas13 not only cleaves the target RNA (red) *in cis* but also cuts other nearby RNAs (blue) *in trans*.

Initial studies of Cas13 in mammalian cells and plant cells have shown comparable levels of knockdown as RNA interference (RNAi) and with improved specificity [12]. While RNAi is less effective against nuclear RNAs, Cas13 efficiently inhibits both nuclear and cytoplasmic RNAs. For instance, Cas13a has been used to knock down nuclear noncoding RNAs such as *MALAT1*, *HOTTIP*[11] and enhancer RNAs [13]. Cas13a has also been used to inhibit cancer-associated fusion transcripts, such as the *EML4-ALK* fusion transcript to inhibit cell viability in lung cancer cells [14]. In addition to linear RNAs, Cas13d can downregulate circular RNAs (circRNAs) by using guide RNAs that target sequences spanning back-splicing junction sites of circRNAs [15]. The flexibility in targeting essentially any sequence without the restriction of PAM/PFS allows most circRNAs to be targeted without affecting the linear transcripts. In addition to endogenous cellular RNAs, Cas13 has been used to degrade viral RNAs, including SARS-CoV-2 [16], influenza virus [16], HIV [17], PRRSV [18] and HPV [19].

Cas13 has also been widely used *in vivo*, allowing targeted RNA knockdown in xenograft tumours [20,21] and various tissues, including eye [22–24], ear [25,26], brain [22,27,28], lung [29–31], kidney [30], liver [30] and spleen [30]. Cas13 proteins, and especially Cas13d proteins, are significantly smaller than Cas9, allowing efficient package into Adeno-associated viruses (AAVs) along with its gRNA expression cassette for *in vivo* delivery [11]. AAV-mediated expression of Cas13a/gRNA targeting several oncogenes significantly inhibited tumour growth in xenograft models [20]. Similarly, AAV-Cas13d targeting the oncogenic *KRAS*^*G12D*^ mRNA significantly inhibited tumour growth in patient-derived xenografts in mice [21]. In addition to tumour, AAV-Cas13d has also been delivered to specific tissues to knock down various targets and achieved phenotypic rescue in mouse models of age-related macular degeneration [23], hearing loss [25], Parkinson’s disease [22], TDP-43 proteinopathy [32] and amyotrophic lateral sclerosis (ALS) [33], respectively. In addition to AAV, non-viral approaches including lipid nanoparticles (LNPs) [31], extracellular vesicles (EVs) [30] and nebulizer-based nanoformulated RNA complex delivery [29] have also been used to deliver Cas13/gRNA, especially to the lung.

## Engineering RNA with CRISPR-dCas13

**Figure 2.**
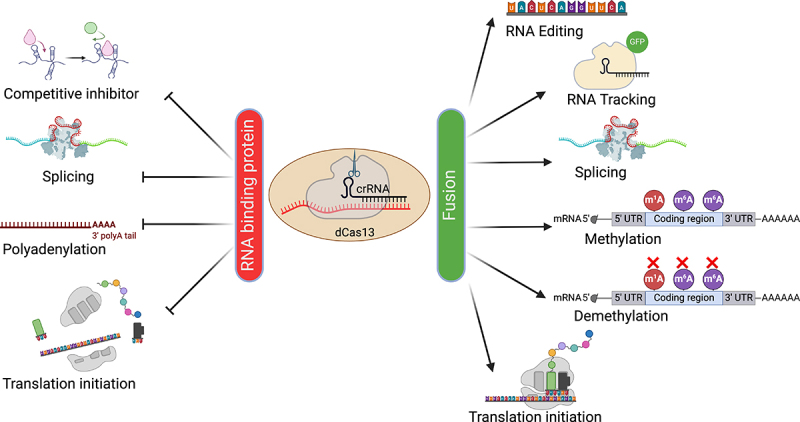
Applications of dCas13.

Point mutations in the two HEPN RNase domains inactivate the gRNA-dependent target cleavage activity, allowing target RNAs to be bound without being degraded (Figure 2). While often called catalytically dead Cas13 (dCas13), the resulting HEPN-deficient Cas13 retains the pre-crRNA processing RNase activity and thus remains capable of multiplexing. dCas13 can be used as a programmable RNA-binding protein (RBP), allowing it to act as a competitive inhibitor of endogenous RBPs on a specific transcript [34]. Similarly, other functional RNA signals such as splice sites, polyadenylation signals and start codons of a specific transcript can be targeted by dCas13 to modulate splicing [11], polyadenylation [35] and translation initiation [36], respectively.

dCas13 alone or its fusion with various effector domains allows precise manipulation of RNAs in eukaryotic cells.

Furthermore, various effector domains have been fused with dCas13 for additional applications. For example, dCas13-GFP has been used to label and track RNAs in live cells [12,37]. Affinity purification of tagged dCas13 loaded with specific gRNAs allows the detection of proteins bound to an RNA of interest [38,39]. Artificial splicing factors fusing dCas13 with domains of splicing factors can modulate alternative splicing of endogenous transcripts [11,40]. Direct insertion or replacement of thousands of nucleotides in endogenous RNAs is now possible with dCas13-mediated trans-splicing [41]. Targeted A-to-I and C-to-U RNA editing have also been developed by fusing dCas13 with either endogenous or evolved deaminase domains, respectively [42,43]. Fusing nucleus- or cytoplasm-localized dCas13 with a methyltransferase domain enables site-specific N^6-^Methyladenosine (m^6^A) incorporation within distinct cellular compartments [44]. Conversely, RNA modifications such as m^6^A and m^1^A can be removed in site-specific manner by fusing dCas13 with corresponding demethylases, such as ALKBH5 and ALKBH3, respectively [45,46]. Translation of specific mRNAs can be enhanced by dCas13 fused to translation initiation factors such as IF3 in *Escherichia coli* [47] and PABPC1 in various human cells [48].

In addition to fusing protein effector domains to dCas13, new functions can be generated by fusing functional RNA elements to the gRNA. While similar ideas have successfully been used in dCas9-based applications [49,50], tethering RNA regulatory elements via gRNAs may be particularly suitable for RNA-targeting applications. For example, the SINEB2 RNA element has been fused with gRNA to enhance translation of target mRNAs [51]. Given the large number of functional RNA regulatory elements controlling every step of the RNA life cycle [52], we envision further expansion of the dCas13 RNA toolkit by tethering other RNA elements to the gRNA. Compared to protein effector domains, RNA elements are typically much smaller, making it easier to multiplex and package in adeno-associated virus (AAV) for *in vivo* delivery.

## Target specificity of Cas13: off-targets and mismatch tolerance

In general, CRISPR/Cas13 exhibits tolerance for a single mismatch between the target RNA, with the gRNA spacer nucleotides 15–21 being more sensitive to mismatches in the target site [53]. The presence of more than two mismatches often leads to a reduction in cleavage efficiency [54], although in some cases more mismatches can be tolerated [55]. Moreover, the sensitivity to mismatches is also highly variable across positions within the gRNA/target RNA duplex as well as the nucleotide identity of the mismatch [56,57]. The lack of a set of simple rules for targeting specificity calls for sophisticated machine learning models to be trained using large-scale experimental measurements of mismatch tolerance for each Cas13 system. Encouragingly, at least two approaches have been shown to decrease mismatch tolerance and allow specific detection of SNPs. The first approach is synthetic mismatch, i.e. use gRNAs with one or more mismatches to the desired targets [58,59]. Cas13 will tolerate the limited number of mismatches in the target but not more mismatches found in off-targets. The second approach is to extend the spacer at the 3’ end of the gRNA with a sequence that will form a short stem loop/hairpin with the spacer. By blocking part of the spacer, the hairpin prevents the gRNA from binding to off-target RNA sequences and improves the specificity of the CRISPR/Cas13a system for single nucleotide polymorphism (SNP) identification [60]. Despite extensive research endeavours, enhancing the specificity of Cas13 systems remains an area that warrants further attention and improvement.

## Cas13 collateral activity in bacteria and in vitro

Upon target RNA recognition, Cas13 undergoes a conformational change and becomes an activated RNase. Unlike most Cas proteins that cleave within the guide/target duplex, the activated RNase site is exposed on the surface of Cas13 [7,8], allowing it to cleave not only the bound target RNA *in cis* but also other nearby RNAs *in trans* (Figure 1). Such collateral activity allows bacteria to degrade both phage RNAs and host RNAs upon phage infection, resulting in growth arrest of the host cell and the abortion of the infectious cycle [61]. Similar ‘suicide’ responses are also triggered by several other antiviral systems [62], including the type IIIA CRISPR-Cas system employing the RNase Csm6 that indiscriminately degrades both viral and host RNAs.

It is important to keep in mind that collateral activity is fundamentally different from conventional off-target effects. Off-targets of CRISPR or RNAi still directly pair with the guide RNA or siRNA, albeit with one or more mismatches. In contrast, Cas13 collateral activity is indiscriminate, and the degraded substrate does not need to have sequence complementarity to the guide RNA. Moreover, off-target effects are largely independent of whether the intended on-target is present, whereas collateral activity is only activated upon recognition of the on-target RNA that perfectly matches the guide RNA, although in principle conventional off-targets can also activate collateral activity, as long as the mismatch is tolerated.

**Figure 3.**
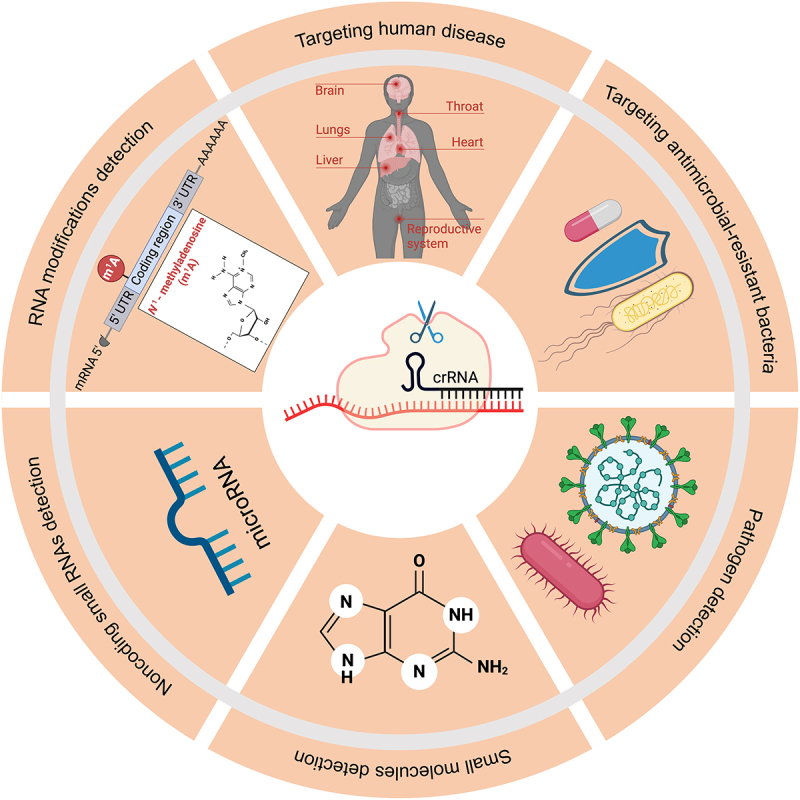
Applications of Cas13 collateral activity.

The collateral activity of Cas13 has found diverse applications across numerous fields (Figure 3). For example, the ability to sense a specific sequence and induce self-destruction of bacterial cells has been used for sequence-specific bacteria killing. By packaging programmed CRISPR-Cas13a recognizing resistance genes into bacteriophage capsids, Kiga and colleagues created a new type of antibacterial agent called ‘CapsidCas13a(s)’ that inhibited the growth of carbapenem-resistant *Escherichia coli* and methicillin-resistant *Staphylococcus aureus*, two types of bacteria known for their resistance to common antibiotics [63]. Supporting a role of Cas13 collateral activity in causing the observed growth arrest, targeting the same gene with CRISPR/Cas9 did not result in the same defect, which is not known to have collateral activity.

Cas13 collateral activity has been used for nucleic acid detection, disease targeting and several other applications.

Cas13 collateral activity has also been leveraged for sensitive detection of RNA or DNA (when coupled with *in vitro* transcription). In the presence of a target RNA, activated Cas13a cleaves a self-quenched reporter RNA, which results in the emission of fluorescence. The amount of fluorescence emitted is proportional to the concentration of the target RNA. The sensitivity can be further increased by isothermal amplification of the target and by incorporating Csm6, an auxiliary CRISPR-associated enzyme [64]. The resulting SHERLOCK platform allows ultrasensitive detection of pathogenic virus (e.g. Zika and Dengue viruses) and bacteria and is also highly specific in distinguishing specific viral strains, human SNPs and tumour mutations [58]. Technical improvements in several related platforms, including HUDSON [65], SHINE [66], CASCADE [67], RT-LAMP-CRISPR-Cas13a [68], have allowed rapid, instrument-free and extraction-free detection of RNAs including SARS-CoV-2 RNA from unextracted samples. Integrated with the CARMEN platform that can simultaneously analyse over 4,500 crRNA-target pairs within a single array, CARMEN-Cas13 can detect all 169 viruses associated with humans in a single test [69]. Cas13 has also been used to detect microRNAs [70,71], RNA modifications [72], as well as small molecules when coupled with riboswitches [73].

## Cas13 collateral activity in eukaryotic cells

While evidence supporting Cas13 collateral activity *in vitro* and in bacterial systems has been abundant and clear, the extent of such activity in eukaryotic cells remains a subject of inconsistency in various published papers. Early studies of Cas13a and Cas13d did not report any off-target or collateral effects in mammalian cells [11,12,42]. Instead, these studies show that Cas13-mediated transcript knockdown is remarkably specific, with zero off-targets compared to hundreds for shRNA-mediated knockdown [11,12] when assayed with RNA-seq. In Drosophila transgenic cell line Sg4_CD, a number of Cas13 variants can efficiently knock down an eCFP reporter without affecting a co-expressed DsRed reporter [74]. Another study tested Cas13d-mediated transcript knockdown in zebrafish embryos and observed no toxic effects, off-target impacts or collateral activity [75]. Similarly, Cas13a downregulated endogenous *PPIB* and *KRAS* mRNAs in human lung epithelial cells A549 with no significant changes in the expression of 11 housekeeping genes and 18S rRNA [29].

More recent studies, however, have found substantial collateral activity of various Cas13 systems in mammalian and *Drosophila* cells using a variety of assays [6,76–79]. For example, we and others have shown that targeting either reporter mRNAs (e.g. EGFP) or endogenous RNAs (e.g. ACTG1) can result in the degradation of co-expressed non-target reporter RNAs (e.g. mCherry), Cas13 mRNAs and other endogenous RNAs, as measured by qRT-PCR, Northern blotting, Western blotting and fluorescence imaging. Multiple studies have also reported a loss of RNA integrity, as indicated by rRNA cleavage and fragmentation in denaturing RNA gel or BioAnalyzer runs [78]. Supporting global RNA degradation caused by Cas13 collateral activity, we further showed a 46% decrease in total RNA extracted from Cas13 targeted cells [77]. Moreover, using polyA+ RNA-seq with external spike-in, we have shown that almost the entire transcriptome is downregulated by Cas13 collateral activity, including GAPDH, ACTB and other housekeeping genes [77].

How do we reconcile the contradictory observations regarding Cas13 collateral activity in eukaryotic cells? The difficulty for internal control-based normalization has probably contributed to the failure to detect collateral activity in some studies, because commonly used control such as GAPDH and even rRNA are also down-regulated by Cas13 collateral activity [77]. Moreover, recent studies have shown that the extent of collateral activity is strongly correlated with target RNA abundance [76,77,79], and earlier studies reporting a lack of collateral activity were mostly targeting low abundance RNAs [11]. There is also a substantial variation of collateral activity observed for different subtypes of Cas13 proteins, with Cas13d showing the strongest effect, followed by Cas13a and then Cas13b [79]. Intriguingly, the extent of collateral activity also varies across cell types. For example, it has been shown that Cas13a exhibits a stronger collateral activity in U87 cells but not in HEK293T cells [78], although we have observed very strong collateral activity in HEK293T cells using Cas13d [77]. Such substantial variations could be due to variation in the expression level of Cas13, gRNA and the target RNA in those cells, or even variation in cellular pH [80–84], as RNase activities are sensitive to pH [85]. Therefore, the extent of collateral activity observed depends on a variety of factors, potentially underlying the contradictory results reported in the literature.

The unintended indiscriminate degradation of cellular RNAs is a major concern for using Cas13 to knock down specific target RNAs in cells, especially for abundant targets. While reducing Cas13/gRNA abundance [86] or stability [76] can potentially reduce off-target binding and thus off-target-activated collateral activity, it is inherently challenging to reduce on-target-activated collateral activity without compromising on-target knockdown efficiency, as the same active site is responsible for both activities. Nonetheless, the collateral activity varies dramatically across Cas13 subtypes (e.g. PspCas13b vs RfxCas13d) and novel Cas13 orthologs (e.g. DjCas13d) with minimal cellular toxicity have recently been identified [87]. Moreover, high-fidelity variants of Cas13d and Cas13X have also been engineered [88], and in particular the RfxCas13d-N2V7 variant has been independently shown to be more specific than Cas7–11, another RNA-targeting CRISPR system not known to have collateral activity [32,89]. It remains unclear mechanistically how collateral activity is modulated in those Cas13 variants/orthologs. Possibilities include structural changes that position the active site closer to the target region and less exposed on the surface, or decrease the stability of the active Cas13-gRNA-target ternary complex, such that it stays active long enough to cleave the target RNA which is in close proximity, but not long enough to cleave a larger number of other RNAs in cells.

## Sequence-specific cell targeting with CRISPR-Cas13

The activation of Cas13 collateral activity arrests the growth of infected bacteria. Similarly in human cells, Cas13-mediated targeting of reporter mRNAs or non-essential endogenous mRNAs substantially reduces the viability and proliferation of cells [77]. Similar to collateral RNA degradation, the extent of growth defect is positively correlated with target RNA abundance [77]. While apoptosis is observed in some studies [76], others reported no increase in cell death, only a reduction in cellular metabolic activity and DNA replication [77]. Interestingly, similar to RNase treatment of nuclei [90,91], Cas13 collateral activity also results in the collapse of chromatin, potentially contributing to global mRNA downregulation and inhibition of DNA replication. The toxicity of Cas13 collateral activity has also been observed *in vivo*. Strikingly, Cas13d-mediated knockdown of several non-essential mRNAs in adult mouse brain resulted in animal death [92]. The lethality strictly depends on simultaneous expression of Cas13, gRNA and the target, suggesting the toxicity is driven by collateral activity rather than off-target effects [92].

The ability to inhibit cell growth or even induce cell death by sensing a marker RNA opens the door for sequence-specific cell targeting. Programmable elimination of pathogenic cells such as cancer cells, activated fibroblasts and senescent cells will facilitate the development of therapies against cancer, fibrosis and ageing, respectively. Such a technology platform will also allow functional studies of novel cell types or cell states uncovered by single-cell RNA sequencing. As a proof-of-principle, we have previously demonstrated in a competitive growth assay that a subset of cells can be selectively depleted by Cas13-mediated sensing of a dispensable reporter RNA uniquely expressed in the target cell population [77]. The same concept has been applied *in vivo* for selective elimination of cancer cells by targeting cancer-specific oncogenic mRNAs. For instance, Kang and colleagues showed that Cas13a targeting of oncogenic EGFRvIII mRNA significantly inhibited tumour growth *in vivo* [78] (although it remains to be shown that Cas13 collateral activity, rather than the loss of the oncogenic target mRNA alone, drove tumour regression). Further enhancing Cas13 collateral activity will enable more potent cell killing. Encouragingly, Yang and colleagues have shown that the collateral activity of Cas13a can be enhanced by inserting an extra RNA-binding domain into a unique active-site-proximal loop within its HEPN domains [93].

## Outstanding questions

Given the numerous applications of the Cas13 system in biomedical research, clinical diagnostics and potential therapeutic applications, it is critical to understand its limitations and avoid potential pitfalls. While high-fidelity variants of Cas13 alleviate the concern of unintended collateral RNA degradation for targeted RNA knockdown, recent studies have uncovered unexpected effect of expressing either Cas13 or gRNA alone. A deeper understanding of these observations and the underlying mechanisms will facilitate the development of next-generation high-fidelity Cas13 tools for various applications.

*Cas13-independent effects of guide RNAs*. While gRNAs are thought to be loaded into Cas13 first and subsequently bind target RNAs via sequence complementarity, the gRNA/target duplex is long (20–30 bp) and can be stably formed in cells even in the absence of Cas13. Binding by the gRNA alone can potentially block regulatory sites on the target mRNA or recruit double-strand RNA (dsRNA) binding proteins. For example, when used in plants, Cas13 gRNAs with 28-nt but not 20-nt spacers down-regulated target RNAs via endogenous RNAi machinery in the absence of Cas13a [94]. Such effect was also seen with Cas9 gRNAs with 28-nt spacers. A similar observation was also made in human A459 cells: one out of three gRNAs tested decreased CXCR4 mRNA by over 90% in the absence of Cas13a [29], although in this case it was unclear whether a long spacer was used and whether the RNAi machinery is involved. Similarly, expressing Cas13b gRNAs with a 31-nt spacer degraded a viral RNA in mosquito cells in the absence of Cas13b protein [95]. Such Cas13-independent RNAi-like target silencing could potentially result in more off-target effects for targeted knockdown and complicate the interpretation of dCas13-based applications such as imaging and splicing modulation. It has also been shown that when the spacer region is longer than 40-nt, the long duplex formed with the target RNA will trigger efficient target RNA editing by recruiting endogenous RNA editing enzyme ADAR1 [96]. The edited target RNA may no longer trigger Cas13-mediated cleavage, potentially explaining why gRNAs longer than 30-nt results in less efficient knockdown [53]. These Cas13-independent effects of gRNAs highlight the caveat of using Cas13 gRNAs with long spacers and underscore the importance of proper controls. More systematic studies are needed to understand the prevalence of Cas13-independent effect and how to avoid it, for both the widely used Cas13 orthologs and the high-fidelity variants.

*Guide RNA-independent Cas13 activity and toxicity*. Several studies have reported unexpected toxicity when certain variants of Cas13 are expressed alone without corresponding gRNAs. For example, plasmid-based expression of LwaCas13a or PspCas13b but not RfxCas13d (CasRx) alone inhibited neurite and dendrite growth in primary cultures of mouse neurons [97,98]. Similarly, injecting PguCas13b and PspCas13b but not RfxCas13d proteins impaired zebrafish embryonic development [75]. While RfxCas13d/CasRx is less toxic in cultured mouse neurons, ubiquitously expressed CasRx caused embryonic lethality in flies, as flies homozygote for CasRx cannot be generated [99]. Intriguingly, even dCasRx homozygous flies cannot be generated. This observation suggests that the observed toxicity is likely caused by the pre-crRNA processing RNase activity, which remains intact in dCas13 [100,101]. Cas13/dCas13 proteins recognize the direct repeat region in pre-crRNAs, which forms a short hairpin with moderate sequence specificity. Similar hairpin structures could potentially be found in many endogenous RNAs in eukaryotic cells, especially in neurons that are known to enrich for dsRNAs [102]. The recognition of these direct repeat-like structures will result in the cleavage and degradation of endogenous transcripts, potentially causing cellular toxicity. Supporting this idea, Li and colleagues discovered that LwCas13a, PspCas13b and RfxCas13d all bind thousands of endogenous mRNAs in HEK293T cells and are capable of cleaving endogenous RNAs *in vitro* without corresponding gRNAs [103]. Future studies will delineate whether Cas13/dCas13 processes endogenous RNAs into crRNAs/gRNAs, and whether inactivating the pre-crRNA processing activity will abolish gRNA-independent toxicity.

## Conclusions

Since its initial discovery in 2016, the RNA-targeting CRISPR/Cas13 system has not only transformed the basic research of RNA biology but has also significantly advanced RNA-based diagnostics and therapeutics. The ability to target essentially any sequences in RNA, nuclear or cytoplasmic, in a highly efficient and specific manner, makes Cas13 a powerful tool for gene targeting. Its compact size, coupled with its inherent multiplexibility, further enhances its potential as a therapeutic platform. The ever-expanding dCas13 toolkit facilitates a diverse range of RNA manipulations with single-nucleotide precision and specificity. The capability to precisely edit endogenous RNAs, including multi-kilobase replacements or insertions, without altering DNA, opens the door for developing safer therapies across a spectrum of diseases [41]. The collateral activity of Cas13, while initially perceived as a constraint, has proven immensely successful in pathogen detection, and has the potential to become a powerful platform for precision cell targeting. We envision continued development of Cas13 technologies to enable more exciting applications in biomedical research and RNA-based diagnostics and RNA-targeting therapeutics.
